# “The leading role of pathology in assessing the somatic molecular alterations of cancer: Position Paper of the European Society of Pathology”: letter to the Editor

**DOI:** 10.1007/s00428-020-02898-2

**Published:** 2020-08-06

**Authors:** Winand N. M. Dinjens, Marjolijn J. L. Ligtenberg, Tom van Wezel, Ed Schuuring, Hendrikus Jan Dubbink

**Affiliations:** 1 Department of Pathology, Erasmus MC Cancer Institute, University Medical Center Rotterdam, P.O. Box 2040, 3000 CA Rotterdam, The Netherlands; 2 Department of Pathology, Radboud University Medical Center, Nijmegen, The Netherlands; 3 Department of Human Genetics, Radboud University Medical Center, Nijmegen, The Netherlands; 4 Department of Pathology, Leiden University Medical Center, Leiden, The Netherlands; 5 Department of Pathology, University Medical Center Groningen, University of Groningen, Groningen, The Netherlands

**Keywords:** Molecular pathology, Molecular diagnostics

To the Editor:

In a recent *Virchows Archiv* publication, Matias-Guiu et al. [1] indicate the central role for pathology in oncology molecular diagnostics. Motivated by this publication, we would like to make several comments.

First, the authors indicate that molecular results must be interpreted in light of morphology and location of the lesion. However, interpretation of molecular results in relation to tumor genetic concepts and molecular (oncogenic and resistance) pathways is also crucial. This accounts not only for molecular analyses of tissues but also of circulating tumor DNA (ctDNA) from liquid biopsies.

Second, as stated, molecular pathology is different from other laboratory specialties because of the oncological pathology specific situation as the use of routine pathology specimens (FFPE, cytology) composed of a mixture of neoplastic and normal cells, and from which often only minute amounts of largely degraded nucleic acids can be retrieved. This pathology-specific molecular biological situation was recognized by the Dutch Pathological Society which implemented since 2014 a 2-year educational program to become a Clinical Scientist in Molecular Pathology (CSMP) [2]. Furthermore, the Dutch Pathological Society established the guideline that pathology laboratories performing molecular pathology should have access to a registered CSMP.

Third, we agree that molecular results should be part of state-of-the-art integrated diagnostics. For securing accredited quality and cost-effective diagnostics, regional centralization of molecular pathology will be inescapable. Ideally, pathologists and CSMPs take the lead in this regionalization and do not leave this to hospitals, insurance companies, or governmental bodies. Within such a regionalized situation, it is important that all pathologists within a region have easy access to the molecular pathology laboratory for consultation and education. In addition, regional molecular tumor boards (MTBs), discussing complex molecular results in the context of clinical and pathological data, should be easy accessible (virtual) for all pathologists, CSMPs, and clinicians.

Fourth, the publication is a plea to perform oncological molecular diagnostics within pathology departments. We think indeed that this is crucial for the best patient care. However, solely the plea is not sufficient; this central role in oncology diagnostics has to be *deserved* by pathology departments. This implies that pathologists need to have broad knowledge of molecular pathology: basic molecular biology including its language and tumor genetic concepts and pathways including targeted treatment resistance mechanisms. In addition, pathologists and CSMPs, in close contact with oncologists, need to keep up with evolving carcinogenesis concepts, implementing new molecular markers, up-to-date molecular technologies, and treatment options.

As a result of the above, it is clear that the pathologist of the future (and current!) has to be a molecular pathologist. Therefore, we would like to plea for implementation of a substantial part (20–25%) of pathology resident training programs to molecular pathology. These molecular pathologists, together with CSMPs, will play a crucial role in translating morphological and molecular features of tumors to diagnostic and predictive parameters that facilitate optimal personalized treatment. As a result, we expect that the leading role of the discipline Pathology in assessing somatic alterations in cancer will be broadly recognized and assigned.
